# Implementing systems- and organisational-level change to advance gender equality in healthcare leadership: a mixed-methods implementation protocol

**DOI:** 10.1186/s43058-026-01030-w

**Published:** 2026-06-26

**Authors:** Belinda Garth, Anusha Ramani-Chander, Jenny Proimos, Darren Rajit, Erwin Loh, Elizabeth Sigston, Graeme Currie, Kathleen Riach, Mariam Mousa, Helena Teede

**Affiliations:** 1 Monash Centre for Health Research and Implementation (MCHRI), Monash University, 43-51 Kanooka Grove, Melbourne, VIC 3168 Australia; 2 Department of Surgery, Monash University, Melbourne, VIC Australia; 3 Warwick Business School, University of Warwick, Coventry, UK; 4 Adam Smith Business School, University of Glasgow, Glasgow, UK; 5 Endocrine and Diabetes Units, Monash Health, Melbourne, VIC Australia

**Keywords:** Systems-level change, Organisational change management, Gender equality, Gender equity, Healthcare leadership, Protocol, Implementation science, Co-production

## Abstract

**Background:**

Despite comprising over 70% of the global healthcare workforce, women remain significantly underrepresented in healthcare leadership. This imbalance has implications for care quality, as leadership diversity is increasingly recognised as a determinant of care quality, equitable outcomes, and health system performance. Structural barriers persist across academic medicine, health services, and professional organisations, limiting career progression and leadership opportunities for women. Existing efforts have largely prioritised individual-level interventions, with limited attention to the systems- and organisational-level determinants that influence adoption, implementation, and sustainability of gender equity initiatives. Coordinated action is needed to address gender inequality through sustainable, evidence-informed systems change.

**Methods:**

This protocol outlines the Organisational Change Management workstream within the Australian Advancing Women in Healthcare Leadership initiative, a nationally implemented, multi-sector partnership. Using a mixed-methods, co-produced implementation research approach, the workstream will implement and evaluate multi-level interventions and associated implementation strategies across system and organisational levels. Guided by the Consolidated Framework for Implementation Research, the Learning Health System framework, and the Reach, Effectiveness, Adoption, Implementation and Maintenance evaluation model, stakeholders are engaged across outer (policy, regulation, funding) and inner (organisational culture, leadership structures) settings. Data collection includes administrative datasets and policy documents, semi-structured interviews, and surveys. Qualitative data will be analysed thematically and quantitative data analysed descriptively. Findings will be triangulated to inform the selection, tailoring, and evaluation of implementation strategies and a codesigned implementation toolkit, supported by iterative learning cycles.

**Discussion:**

This protocol describes a national initiative applying a systems- and organisational-level, co-produced approach to advancing gender equality in healthcare leadership, engaging health services, professional colleges and associations, government, and women in the workforce. By leveraging implementation science and systems change methodologies, the initiative aims to support sustainable organisational transformation. The protocol provides a replicable framework for advancing gender equality in healthcare leadership and beyond and contributes to implementation science by demonstrating how multiple established frameworks can be integrated to operationalise large-scale, system-level organisational change in complex healthcare systems.

**Supplementary Information:**

The online version contains supplementary material available at https://doi.org/10.1186/s43058-026-01030-w.

Contributions of Literature •Demonstrates how multiple established implementation science frameworks – the Consolidated Framework for Implementation Research (CFIR), Learning Health Systems, and RE-AIM – can be integrated to operationalise large-scale, system- and organisational-level change in complex healthcare settings.

•Addresses a gap in implementation science literature by shifting attention from individual-level interventions toward system- and organisational-level determinants, implementation strategies, and sustainability mechanisms.

•Provides a co-produced, mixed-methods protocol for implementing and evaluating multi-level interventions across diverse stakeholder contexts, including health services, professional organisations, and government.

•Illustrates how determinant assessment (CFIR), interactive adaptation (LHS), and structured evaluation (RE-AIM) can be aligned within a single, scalable approach to support sustainable system- and organisational-level transformation.

## Background

Women comprise 70% of the global healthcare workforce, yet remain significantly underrepresented in healthcare leadership [1, 2]. Despite international policy mandates prioritising gender equality – such as the United Nations Sustainable Development Goal Five ‘Achieve Gender Equality and Empower All Women and Girls’, World Health Organization objectives, and the Lancet Challenge [3–5] – progress has been slow. Globally, women hold only 31% of executive director positions and 20% of board chair roles in global health organisations [1], and just 28% of Dean positions in leading public health and medical schools [1, 6]. In academic medicine, women are less likely than men to be promoted to associate or full professor, or appointed to department chair roles [7]. Persistent income disparities further compound these inequities [8]. Promotion and tenure processes have been identified as key contributors to gender disparities in career advancement [9]. These inequities are well documented, including across public and private health services [10], global health institutions [11], academic medicine [12], and leadership within medical colleges and specialty societies [13]. The intersection of gender with race and ethnicity further compounds inequality and marginalises many women from progressing to leadership [14–16].

Globally, progress toward gender equality in workplace leadership remains slow across all sectors [17]. At the current rate of change achieving equal representation is projected to take another 140 years [18], and while not specific to healthcare, this estimate highlights the urgent need for coordinated action, particularly in a sector such as healthcare where women comprise the majority of the workforce [1, 2]. Women in healthcare should not have to wait generations for equitable leadership opportunities. This persistent inequality is not only unjust; it demands immediate and sustained action [17].

Beyond fairness and representation, diverse leadership benefits everyone and has been increasingly recognised for its positive impact on health system performance. Leadership influences governance, strategic decision-making, organisational culture, and priorities that affect healthcare delivery. In workplaces, diverse leadership is linked to a more engaged and motivated workforce, improved retention, and stronger organisational performance [1, 19]. In healthcare systems, increasing the representation of women in leadership is associated with improved quality of care, lower patient mortality, and more equitable health outcomes for patients, particularly for women and children [1, 11, 20]. This positions leadership equity not only as a workforce issue, but as key lever for improving care delivery and health system effectiveness.

Despite the well-established benefits of diverse leadership, structural and systemic barriers to women’s advancement in healthcare leadership persist [21–24]. Progress has been limited, in part, by a historical focus on organisational norms that prioritise individual-level solutions – often placing the onus on women themselves – rather than addressing the broader system- and organisational-level contexts in which they work. To move forward, there is a recognised need to shift the responsibility for change from individuals to collective, system-wide and organisational action [25, 26]. Meaningful and lasting progress requires coordinated, sector-wide strategies that are evidence-based and replace fragmented, adhoc efforts. This involves active engagement, partnership, and co-production with key stakeholders, drawing on cross-sector expertise and centring the voices of women in the workforce at all levels, with particular attention to those in leadership roles, where the greatest inequalities persist [22, 27].

From an implementation science perspective, the challenge is not a lack of evidence on effective gender equity interventions, but *how* to translate this evidence into routine systems- and organisational-level practice at scale.

### The Advancing Women in Healthcare Leadership (AWHL) initiative

It is in this context that the Australian, nationally implemented AWHL initiative was established. AWHL is funded through two competitive National Health and Medical Research Council (NHMRC) Partnership Grants (APP1198561 and 2018718) and aims to partner, co-produce, implement, evaluate and scale multi-faceted system-, organisational- and individual-level interventions to improve career progression to leadership for women in healthcare [28]. A stakeholder matrix guided the establishment of this national partnership, which spans leading health services, professional colleges and associations, and government. The process was guided by cross-sector and international academic expertise. Crucially, the voices of women have been recognised as a collective stakeholder group, with representation across the full career trajectory (from trainees to senior leaders), multiple healthcare settings (including private and public health services and primary care), diverse cultural backgrounds, and a range of locations (from metropolitan to rural). This ensures that interventions are tailored and contextualised to reflect their needs, preferences, and lived experiences; ultimately informing more effective implementation.

### Organisation Change Management (OCM) workstream

The OCM workstream focuses on transforming the systems and organisations in which individuals train and work, with an emphasis on embedding equity processes and practices into the culture of healthcare systems so that change is systemic, collective, and sustainable. The OCM workstream:

- *Aligns individual advancement with systemic reform*: It aims to ensure that women’s progression into leadership is not reliant on exceptional personal effort or resilience, but is instead supported by equitable policies, practices, and institutional norms.
- *Positions equity as an organisational responsibility*: Rather than placing the burden on women to adapt to existing structures, OCM reorients those structures to become more inclusive, adaptive, and reflective of the diverse healthcare workforce.

Within this workstream, the study described in this protocol seeks to co-produce evidence-based, systems- and organisational-level change, in partnership with women in the workforce, researchers, clinicians and leaders, to advance women in healthcare leadership.

## Methods

The primary aims of AWHL’s OCM workstream are to: (1) Identify implementation determinants that influence the adoption, implementation, and sustainability of system- and organisational-level gender equity interventions; and (2) Co-produce, implement and evaluate evidence-based system- and organisational-level interventions, including the implementation strategies required to support adoption, fidelity, adaptation, and sustainability across contexts.

### Background and co-production approach

A multiple-case study design will be used, with the partner organisation as the unit of analysis [29]. Partner organisations have been selected because they represent strategic stakeholders across health services, professional colleges and associations, government, and women in the healthcare workforce. This design enables in-depth exploration of each case within its real-world context, while facilitating cross-case learning.

The AWHL initiative employs co-production-implementation research methods, engaging partner organisations throughout each stage [28, 30, 31]. Strong stakeholder partnerships are foundational to this approach [32]. While the definition of co-production varies, in AWHL it is understood as: stakeholders working together from the outset with shared power and equal partnership, bringing different perspectives including their local context to influence the way interventions are designed, implemented and delivered, to optimise impact on policy and practice [33, 34]. Importantly, solutions are created with active input from those traditionally considered ‘decision-makers’ (e.g., government, member organisations and health service managers) and those traditionally considered on the receiving-end or ‘subjected’ to these solutions (e.g., women in the workforce) [32]. The data collection and analysis process is designed to ensure equitable representation of voices and contributions from individuals across varying positions of power.

This approach facilitates collective action through the construction of a shared agenda [30], activities and actions to implement meaningful, evidence-based and actionable change-focused organisational interventions for measurable impact. This then leads into collaborative implementation of tailored solutions, led by partners and facilitated by academic experts.

The AWHL initiative is an ongoing national program of research and implementation. Foundational activities, including establishment of the partnership and governance structures and an initial program of evidence synthesis (e.g., systematic reviews and meta-synthesis), have been completed or are underway. The implementation research activities described in this protocol represent a prospectively planned workstream.

### Implementation frameworks

**Fig. 1:**
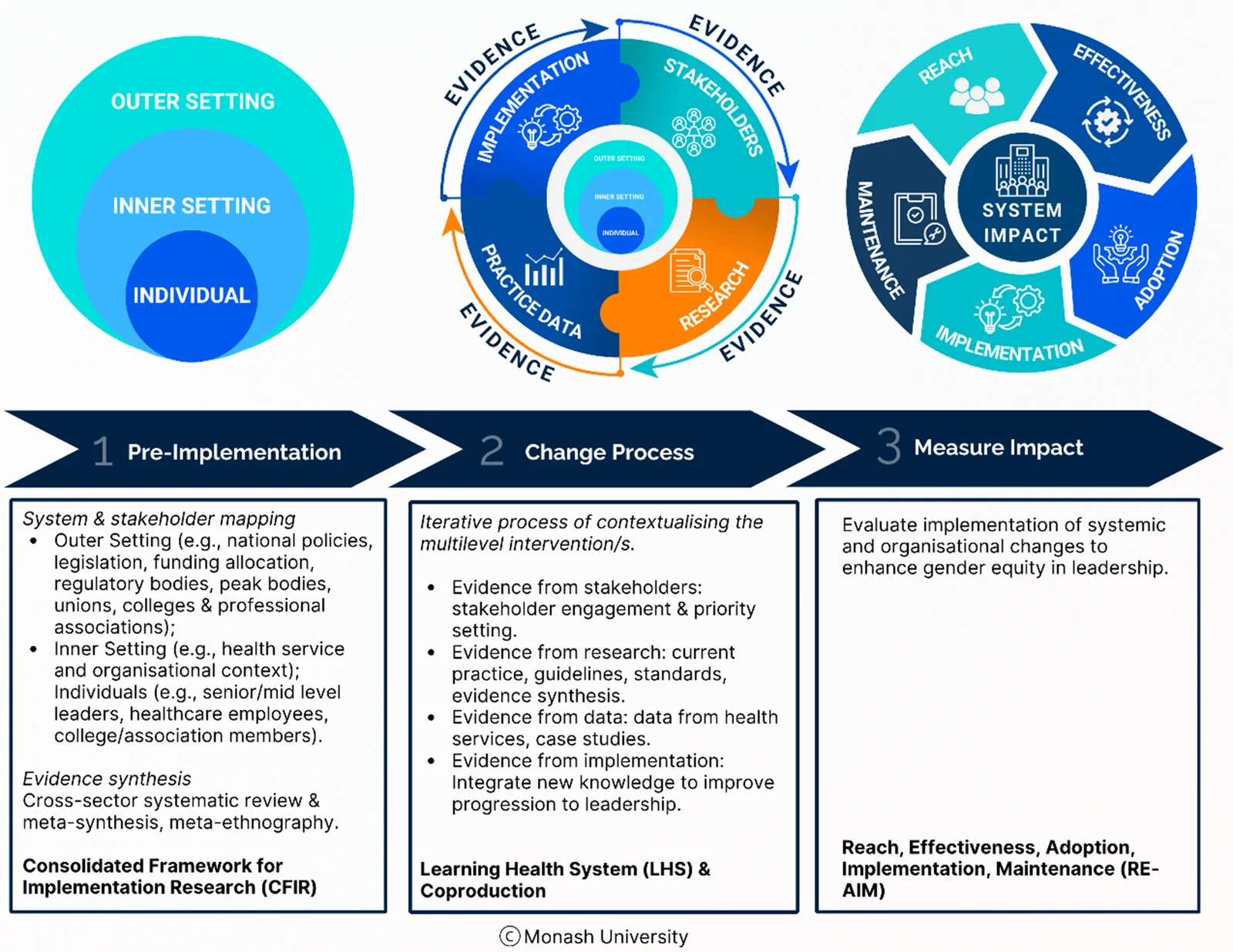
The Monash Systems Change Approach, incorporating the Consolidated Framework for Implementation Research [36], Learning Health System [38, 39] and the Reach, Effectiveness, Adoption, Implementation and Maintenance [40] frameworks

Our approach integrates established implementation science frameworks through what we term the Monash Systems Change Approach, designed to link determinant assessment, implementation strategy selection, iterative adaptation, and outcome evaluation: (1) the Consolidated Framework for Implementation Research (CFIR), (2) the Learning Health System (LHS), and (3) the Reach, Effectiveness, Adoption, Implementation and Maintenance (RE-AIM) evaluation framework, as shown in Fig. 1. A high-level procedural timeline provides an overview of the sequencing of activities and how they interrelate within the broader change process (Table 1).

**Table 1:**
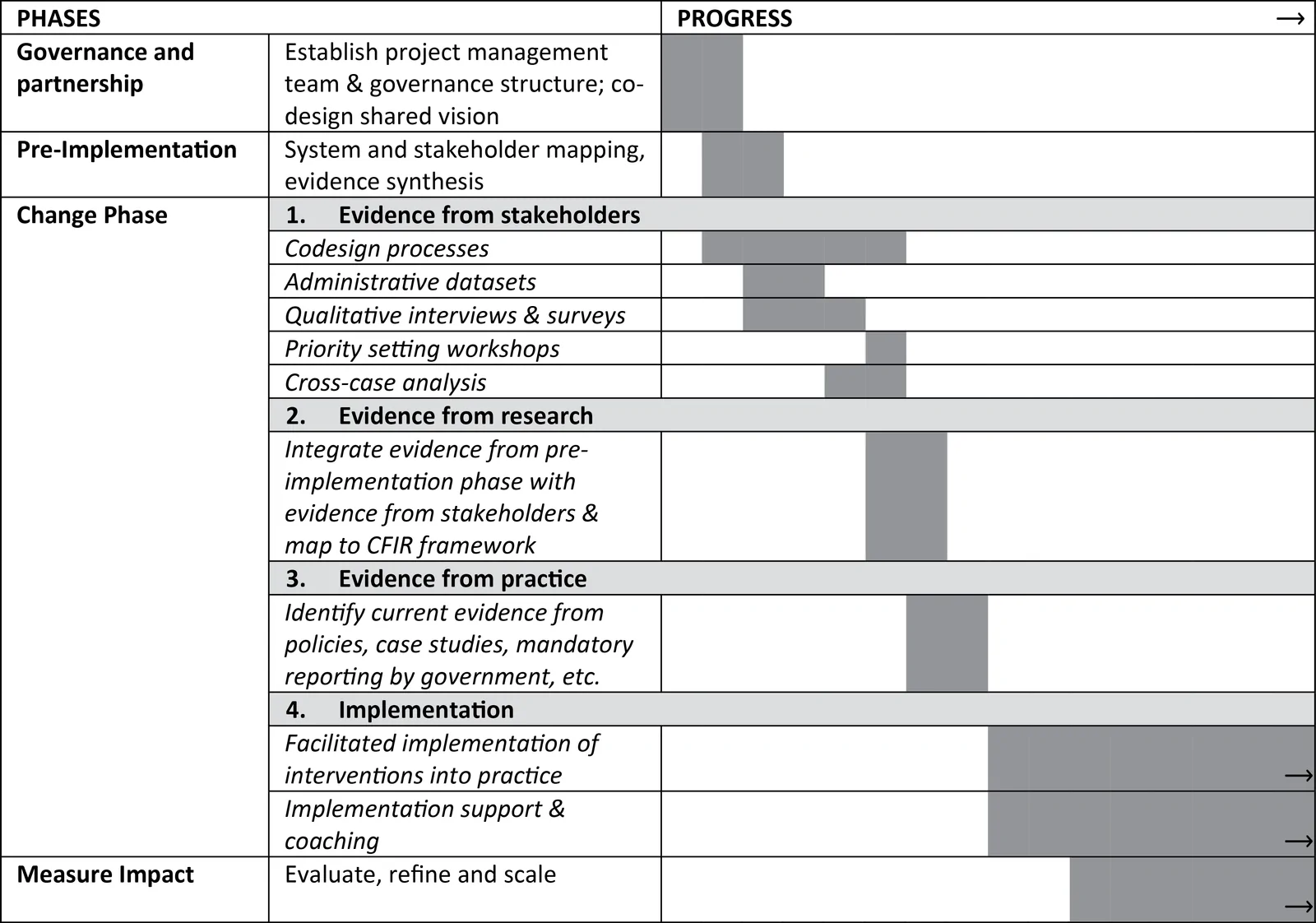
High-level procedural timeline

#### CFIR – to map multi-level determinants of systems change

The CFIR will be used to systematically assess multi-level implementation determinants across outer setting, inner organisational, and individual domains, informing the selection and tailoring of implementation strategies within each partner context [35]. Spanning five core domains, CFIR comprises: (1) the outer context, e.g., government, policy, advocacy, and peak health bodies; (2) the inner organisational context, e.g., the norms, assumptions, culture, values and readiness of health services and academic centres; (3) the individuals in the system, e.g., women in the workforce such as clinicians, nurses, allied health staff, and management; (4) the evidence-based multi-level interventions to be implemented; and (5) the process of implementation.

#### LHS – to enable iterative learning and adaptive systems change

While CFIR offers a robust framework for mapping systems and stakeholders, it functions primarily as a static, determinant framework. This limits its utility in complex systems where contextual factors can shift and evolve over time. CFIR is well-suited to identifying *what* multi-level factors are important (e.g., across the outer setting, inner setting, and individual levels), but it provides limited guidance on *how* to leverage these insights to co-produce context-sensitive strategies [36]. To address this gap, the LHS framework will be applied as the primary mechanism for iterative learning, real-time feedback, and adaptive implementation within dynamic system contexts [37, 38]. The LHS captures, generates and leverages evidence across four broad quadrants, including from stakeholders, research, practice/data and implementation to drive evidence into practice and towards systems and individual behaviour change, in this case for gender equity interventions in health leadership. The CFIR and LHS frameworks are complementary and will be applied to synergise research and implementation efforts.

#### RE-AIM – to evaluate system‑level impact and sustainability

The RE-AIM framework will be applied to evaluate the impact and sustainability of system- and organisational-level changes aimed at enhancing gender equality in leadership [39]. Evaluation will be guided by the RE-AIM framework to assess implementation outcomes including reach, adoption, and implementation fidelity, alongside effectiveness and maintenance outcomes.

While some RE-AIM domains (e.g., reach, adoption, implementation) will be assessed using pre-specified indicators, effectiveness and maintenance outcomes will be co-designed with partner organisations to reflect contextually relevant priorities and system-level goals.

While the primary focus is gender equity in leadership, these measures will also serve as indicators of broader health system capability – reflecting organisational readiness, leadership effectiveness, and capacity to deliver high-quality, equitable care.

### Governance, partner organisations, settings and stakeholders

Led by the Monash Centre for Health Research and Implementation (MCHRI), Monash University, Australia, nine organisations formally partnered to establish AWHL, supported by two academic institutions from the United Kingdom. Partner funding was provided in cash and in-kind contributions, underpinning a successful competitive National Health and Medical Research Council (NHMRC) Partnership Grant. Following this, additional stakeholder partners and another academic institution joined the initiative with extended research themes, leading to a second successful competitive NHMRC Partnership Grant and total funding exceeding $6 million AUD.

Partnerships across leading health networks and professional medical and nursing colleges, government, and academia are now well established, supported by legal multi-institutional agreements, robust governance structures, and shared staff and resources.

**Fig. 2:**
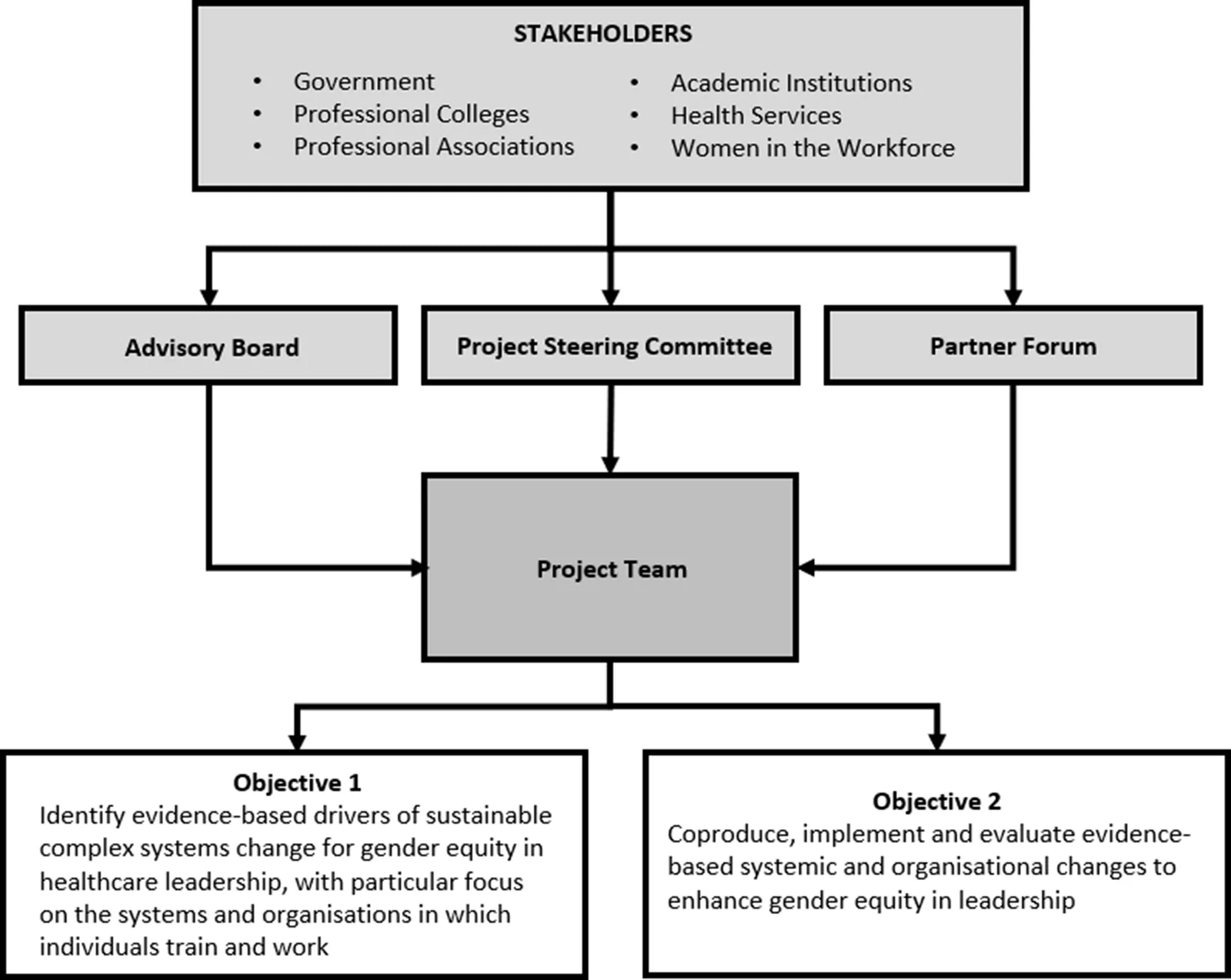
Overall OCM workstream governance structure

Stakeholders and academics with expertise across health, business, and leadership established the project’s leadership and governance structure, including a steering committee to oversee research, implementation and evaluation. An advisory committee was formed to provide strategic guidance and expert advice. A project management team – including the authors – has also been established, bringing expertise in health, leadership, gender equity, and mixed-methods research to conduct the project and partner with stakeholders. The overall governance structure is illustrated in Fig. 2.

AWHL directly engages with the stakeholder groups, brokered, supported and facilitated by academic researchers. Partner organisations include a broad range of stakeholders and settings; public and private health networks (across multiple hospitals and community health services), professional medical and nursing colleges, government agencies, and not-for-profit organisations, including one funded to support equity in the academic sector (Supplementary file 1). The workforce across these member organisations and healthcare networks constitutes a collective of women in the workforce recognised as key stakeholders. We will engage women in the workforce through surveys, qualitative interviews, and workshops, where they directly inform priorities. Engagement will also occur through women in representative roles (e.g., committees) at their organisations serving, as intermediaries between the project team and women in the workforce. A shared vision to advance women in healthcare leadership was developed through a codesign workshop in July 2021 with founding partners. Seven participants attended the online workshop, representing their organisations across policy, executive, governance and board-level roles, and reached consensus on project scope. Participants brainstormed their vision and goals for change, which informed the shared vision, target groups and data collection approaches.

### Data collection tools and analysis

**Fig. 3:**
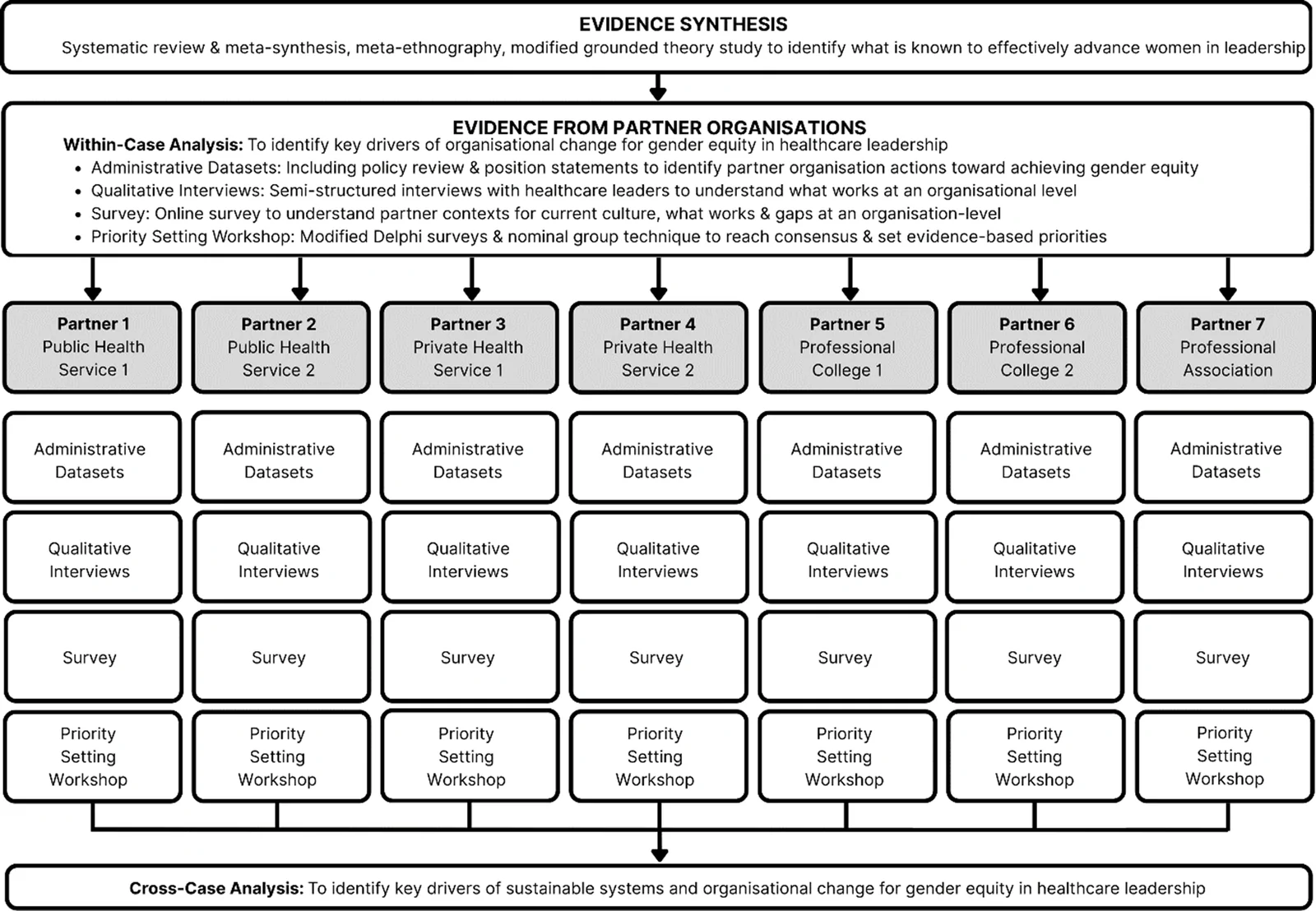
Process for data collection and analysis

A range of data collection tools will be used (Fig. 3). A systematic review and meta-synthesis [26], meta-ethnography [40], and modified grounded theory study [41] have been completed to identify: (i) enablers and barriers to leadership capacity, capability and credibility; (ii) effective interventions and strategies for organisational change to advance leadership for women; and (iii) effective implementation strategies, theories and practical applications, including content and delivery methods. These completed and ongoing evidence synthesis activities inform the design of the prospective data collection, co-production, and implementation phases described below.

Further research with lead partners – each representing a distinct healthcare stakeholder context and constituting a single case [29] – will undergo in-depth exploration through purposeful engagement, to identify organisational strategies, strengths, gaps, opportunities and priorities for achieving gender equality in healthcare leadership. Aligned with our co-production approach, lead partner organisations will be involved from the outset [28]. Partners will work with the AWHL team to develop objectives, data collection tools, and participant recruitment strategies. This active input will continue throughout data collection, analysis, and dissemination to ensure shared findings and iterative improvements.

The three main data collection methods for each case are:

1. *Administrative datasets*. Partner organisation administrative datasets and policy documents will be reviewed to identify past and current initiatives aimed at promoting gender equity and equality in leadership. Information about gender representation across the workforce, training, and governance committees will also be examined.
2. *Semi-structured interviews.* Healthcare leaders will be interviewed to understand partner contexts and identify what works at an organisational level.
3. *Online survey.* Workplace employees and members of professional colleges and associations will be surveyed to understand partner contexts for current culture, identify what works and highlight gaps at an organisational level.

Interview guides and surveys will be co-designed, and questions informed by the Monash Systems Change Approach, evidence synthesis, and stakeholder feedback.

Semi-structured qualitative interviews will be undertaken with sample size guided by appraisal of information power – determined through appraisal of the study aim, sample specificity, use of theory, quality of interview dialogue and analysis strategy to develop new knowledge [42]. Purposive sampling will be used across partners to identify women and men in leadership positions within targeted groups (e.g., across disciplines such as nursing, medicine, allied health; career stages; public and private sectors; culturally diverse groups; health service managers; and policy makers). All interviews will be audio-recorded, transcribed, and anonymised.

Surveys will be administered using Qualtrics software to health service employees and members of professional organisations. All individuals with access to the survey link will be invited to participate. These instruments will generate both quantitative and qualitative data spanning career stages, disciplines, sectors (public and private), and individual versus organisational levels. Response rates and sample characteristics will be reported in full to support assessment of feasibility, representativeness, and generalisability.

Analysis will first occur within each case [29]. Template analysis will be utilised to thematically analyse qualitative data [43]. Template analysis was chosen for its clear, systematic, and flexible approach. A coding template will be developed both inductively and deductively by the project team, guided by the study’s aim, evidence synthesis, Monash Systems Change Approach, while remaining open to new insights [43]. The template will be finalised with input from partners and the project team, and the remaining dataset will then be coded to this template. Quantitative data will be descriptively analysed and summarised.

Findings from administrative datasets, interviews, and surveys will be triangulated within each case and fed back to individual partner organisations to support interpretation and collective sense-making, and identification of strengths, gaps and opportunities.

**Fig. 4:**
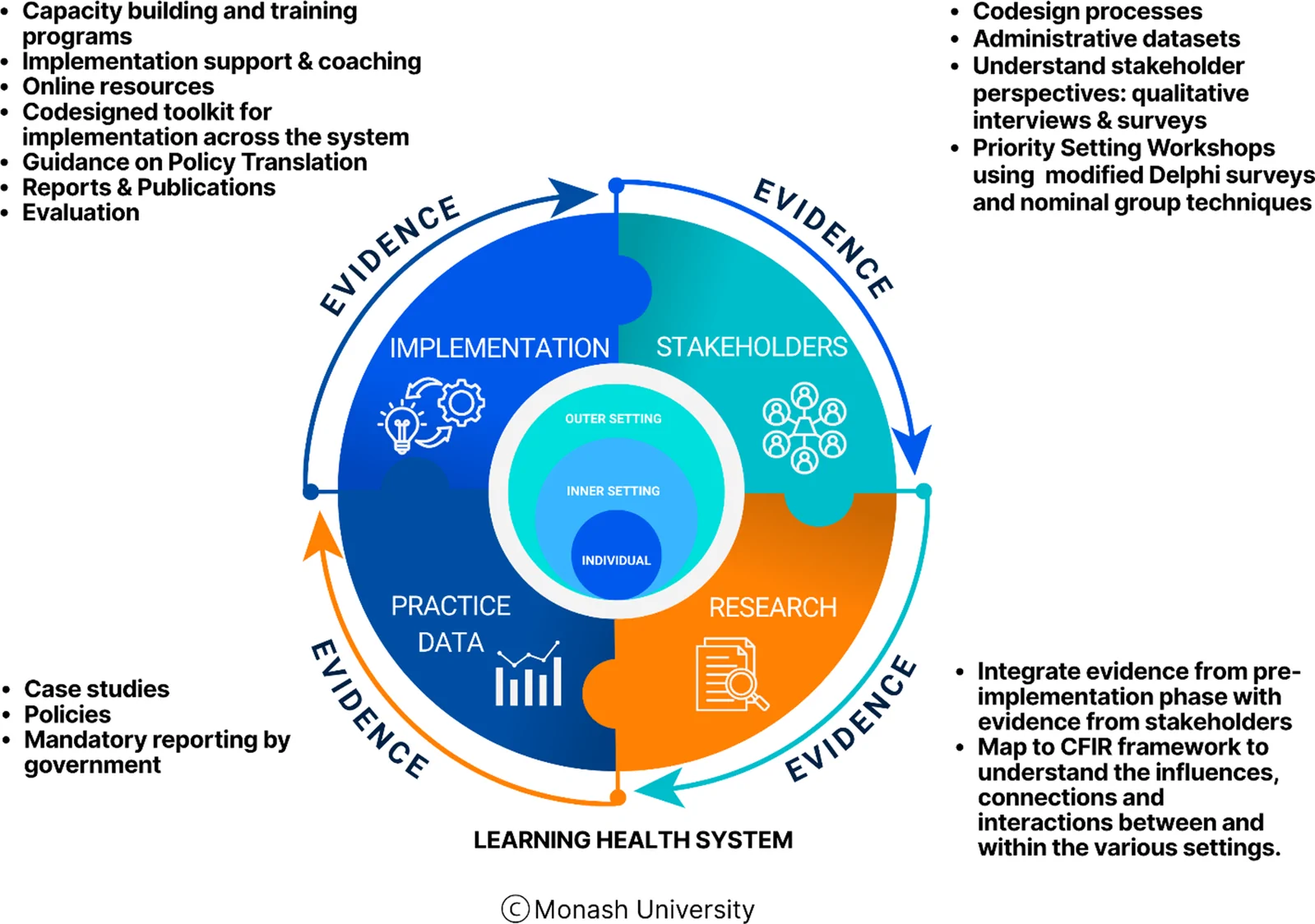
Methodology underpinning the project mapped against the Learning Health System [39]

Once data collection and analysis are complete, each partner organisation will receive a report outlining their results, identifying what is working in their context and opportunity areas to advance women in leadership. A priority-setting workshop for each partner organisation will then be convened, led by the partner organisation and supported by the AWHL team. Modified Delphi surveys and nominal group techniques [44] will be employed to guide a consensus-driven identification of organisational priorities for tailored interventions. The priority-setting process will comprise the following steps:

1. *Participant selection.* Partner organisations will identify and invite leaders and employees or members from across their organisations, ensuring diversity across career stage and gender.
2. *Part One: Pre-workshop ranking survey.* Two weeks prior to the workshop, all employees or members, including workshop participants, will be invited to complete a confidential and anonymous online survey (‘round one’). They will be presented with a list of contextualised evidence-based interventions – identified through the process outlined in Fig. 4 – aimed at delivering gender equality outcomes that advance women into leadership at the organisation. Participants will be asked to rank the interventions they consider most important and acceptable.
3. *Part Two: Priority-Setting Workshop.* Facilitated by the AWHL team, a three to four-hour workshop will include:
  - Overview of the evidence-base, context, and purpose.
  - Open discussion of the Top 10 ranked interventions from the pre-workshop survey.
  - ‘Round two’: Individual, confidential polling to rank the Top 10 interventions by importance.
  - Presentation of round two findings to participants.
  - Introduction of a feasibility framework and discussion of feasibility for the interventions ranked in point c).
  - ‘Round three’: Individual, confidential polling to rank interventions by feasibility.
  - Presentation of collectively agreed priorities to participants.

4. *Action Plan*. The agreed priorities will inform an Action Plan and an implementation and evaluation strategy for each partner organisation to deliver gender equality outcomes. The AWHL team will provide implementation support throughout.

Following the workshop, a report will be compiled and provided to the partner organisation for formal endorsement by its Executive and/or Board. Once context-specific data collection, analysis, and priority-setting have been completed for each partner organisation, a cross-case analysis will be undertaken to identify shared learnings across all partners.

### Methodology

The methodology for the OCM workstream is grounded in implementation science and systems change theories, as outlined in Fig. 4 and discussed in detail below.

### Pre-implementation phase (Aim 1)

The aim of this phase is to identify implementation determinants that influence the adoption, implementation, and sustainability of system- and organisational-level gender equity interventions.

First, comprehensive system mapping activities will be undertaken to engage key stakeholders across both the outer and inner settings, and to maximise engagement and impact across systems levels [35]. This process will identify stakeholders and clarify their roles – an essential step for fostering meaningful engagement and driving organisational change.

Second, evidence synthesis will assemble global and cross-sectoral evidence and map past and current gender equity initiatives to help identify the characteristics of multi-level interventions targeting organisational and systems change.

### Change phase (Aim 2)

Informed by phase one, the aim of phase two is to co-produce, implement and evaluate evidence-based system- and organisational-level interventions, including the implementation strategies required to support adoption, fidelity, adaptation, and sustainability across contexts.

To enhance transparency and reproducibility, the system- and organisational-level interventions and associated implementation strategies will be specified using key implementation parameters, including actors, actions, timing, and mechanisms for tailoring. As a co-produced implementation study, these elements will be refined through stakeholder engagement, determinant assessment, and priority-setting within each partner organisation.

For example, within a partner organisation, actors may include executive leadership, human resources teams, clinical leaders, and the AWHL implementation team. Actions may include co-designing and implementing revised recruitment and promotion policies, delivering leadership development initiatives, and embedding gender equity metrics into organisational reporting systems. Timing will follow the staged approach outlined in Table 1, beginning with stakeholder engagement and priority-setting, followed by action planning, implementation, and iterative evaluation cycles. Multi-component interventions such as three- to four-hour facilitated workshops, ongoing implementation support and coaching, and repeated learning cycles within the Learning Health System framework may also be applied. All interventions and strategies will be tailored to local context using CFIR-informed assessment of organisational determinants and stakeholder priorities. These examples are illustrative and are intended to demonstrate the approach to specification; final intervention content and delivery will be determined through co-production and iterative adaptation within each partner context.

The change phase will be guided by the LHS framework, underpinned by co-production, and will facilitate the process for iterative contextualisation, adaptation, implementation and evaluation of multi-level interventions. The LHS framework supports continuous learning cycles through integration of evidence from four interrelated quadrants: (i) stakeholder engagement and priority-setting, (ii) evidence synthesis and knowledge generation, (iii) current practice and data and (iv) implementation. The quadrants inform one another sequentially, with each drawing cumulatively on those that precede it.

#### Quadrant 1: Stakeholder engagement and priority-setting

The first quadrant of the LHS framework, ‘Stakeholder derived evidence’, emphasises priority-setting and understanding stakeholder perspectives to ensure that developed interventions are relevant and responsive to their needs [38]. Across each lead partner – representing distinct healthcare stakeholder contexts – administrative datasets will be analysed to identify past and current organisational initiatives aimed at promoting gender equity and equality in leadership. Subsequently, interviews with healthcare leaders (approximately 20 per partner) and surveys with employees or members will be conducted and analysed, as previously outlined. Data will be analysed individually for each partner and presented in tailored reports. To support strategic planning, priority-setting workshops will be conducted within each lead partner organisation, facilitated by the AWHL project team. The aim of these workshops is to establish agreed, evidence-based priorities for delivering gender equality outcomes within each partner organisation. Modified Delphi surveys and nominal group techniques [44] will be used to identify consensus-based organisational priorities for advancing gender equality in leadership. Regular meetings with lead partners will be held to present and discuss emerging findings. Oversight will be provided by an established steering committee comprising delegates from each lead organisation.

#### Quadrant 2: Evidence synthesis and knowledge generation

The second quadrant of the LHS framework, ‘Research derived evidence’, focuses on generating knowledge and synthesising findings with existing research [38]. To understand structures and processes required to drive, implement and evaluate systemic and organisational changes to improve gender equality in leadership, evidence synthesis from published literature and knowledge generated from the data collection methods described earlier (administrative datasets, interviews, surveys) will be mapped to the CFIR framework. This mapping will support understanding of the influences, connections, and interactions between and within the various settings.

#### Quadrant 3: Current practice and data

In the third quadrant of the LHS framework, ‘Data derived evidence’ emphasises understanding and integrating current practice and data to support iterative change processes [38]. Research case studies will be informed by the findings outlined earlier. The implementation science and CFIR approaches address systems, organisational and individual perspectives, and hence enable exploration and evidence generation on systems change (e.g., across government, legislation and accreditation standards) and organisations (e.g., who create the workplace conditions and culture that can enable gender equality in leadership). Implementation strategies and resources will be developed to reflect findings and recommendations from semi-structured interviews and surveys that capture current practice in healthcare.

#### Quadrant 4: Implementation and healthcare leadership improvement

The fourth quadrant of the LHS, ‘Implementation evidence’, focuses on translating rigorous implementation research into practice to advance gender equality in healthcare leadership [38]. Key outputs will include a co-designed implementation toolkit, developed using the UK Design Council Double Diamond co-design process. This process fosters co-design through four phases: (i) discover – working with stakeholders to understand the problem; (ii) define – focusing on specific problem areas; (iii) develop – identifying solutions with stakeholders; and (iv) deliver – testing and evaluating solutions [45].

Additional outputs will include a suite of interventions tailored to partner needs, which can be adapted, implemented and scaled. These may include policy translation guidance and policy reform (e.g., equitable recruitment and promotion processes), culture‑change initiatives (e.g., addressing bias and fostering inclusive leadership environments), leadership development and training programs, structural and workflow redesign (e.g., flexible work models, transparent career pathways), and organisational capability‑building to support sustained change. Each intervention will be operationalised with clearly defined actors, actions, delivery mechanisms, and implemented through co-produced strategies such as stakeholder workshops, consensus processes, action planning, and ongoing implementation support.

**Fig. 5:**
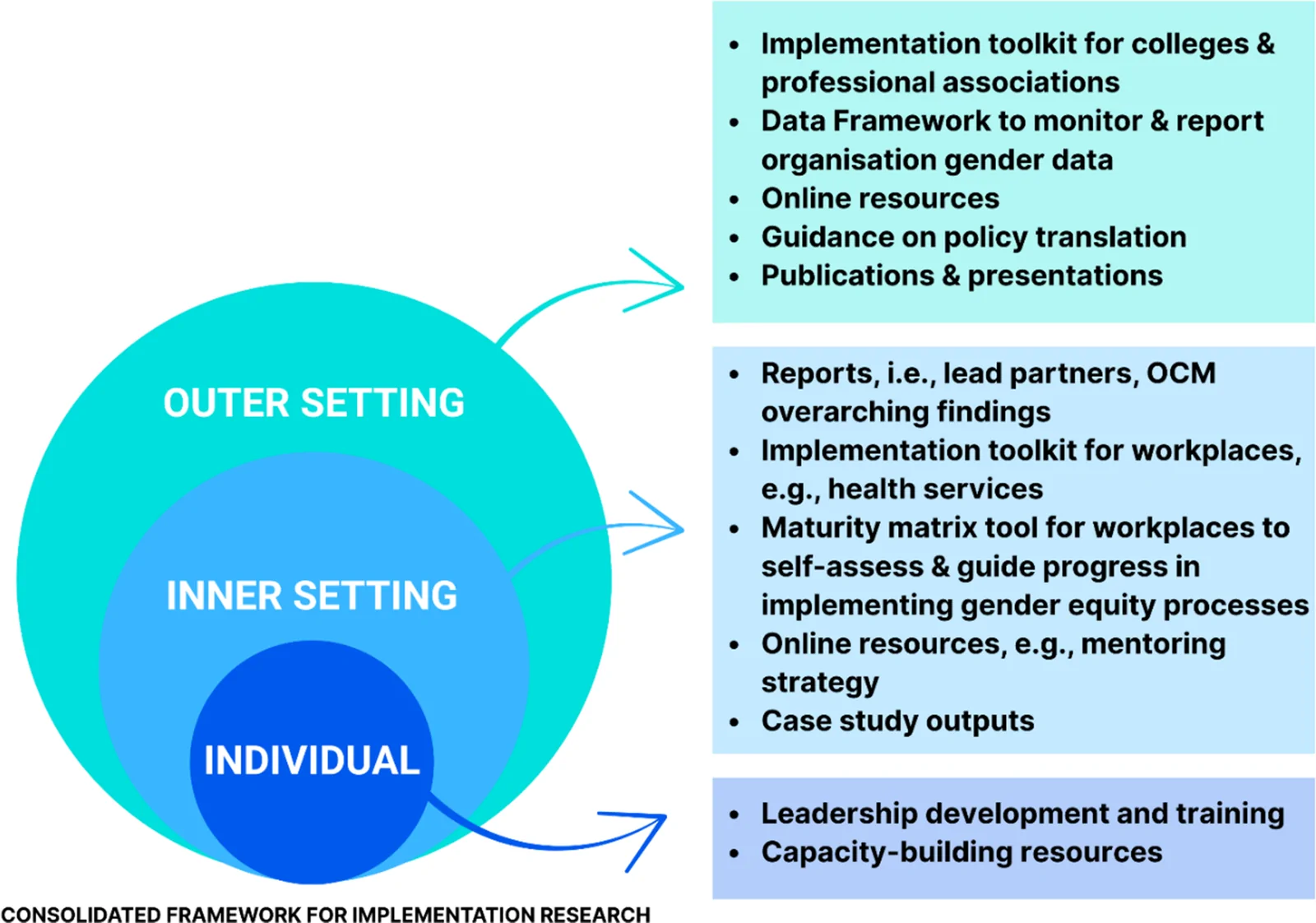
Implementation resource outputs mapped against the Consolidated Framework for Implementation Research [36]

Multi-layered strategies will be mapped to the CFIR framework to enable a targeted approach across each level of the system. Understanding the role of key stakeholders is expected to provide insight into the support and changes required to promote adoption and drive meaningful change, as illustrated in Fig. 5. By strengthening leadership equity at system and organisational levels, these interventions are expected to support improvements in decision-making quality, implementation capacity, and organisational culture – factors critical to the delivery of safe, high-quality, and patient-centred healthcare.

Building on this foundation, the next phase will focus on evaluating the effectiveness and sustainability of these strategies to ensure they translate into meaningful, system-wide change. Evaluation of the implementation toolkit and the multi-layered strategies, designed to support equitable and fair career pathways to leadership, is intended to identify areas for refinement, thereby continuing the LHS cycle.

Implementation and evaluation will be co-designed with partner organisations. Evaluation will be guided by the RE-AIM framework, applied across reach (tool use and uptake), effectiveness (outcomes to be co-designed), adoption (integration into systems and organisational processes), implementation (fidelity of evidence-based processes and tools) and maintenance of outputs over time. In line with implementation science approaches to adaptive and co-produced interventions, the specific configuration and delivery of interventions and strategies will vary across partner organisations and will be finalised following assessment and stakeholder priority-setting. Examples provided here are therefore illustrative rather than prescriptive. This approach ensures that implementation strategies are responsive to contextual variation while maintaining consistency in the overarching methodological framework and evaluation approach.

### Ensuring study quality

The AWHL initiative has undergone independent scientific peer review by the NHMRC under two successful Partnership Project Grants. Study quality and risk management will be monitored and ensured throughout the project by the steering committee and advisory board. The AWHL steering committee meets at least quarterly and provides guidance and advice to the project team to ensure all goals and objectives are met. The advisory board meets biannually and provides ongoing strategic guidance and expert advice to the steering committee and project team. This includes support in developing strong collaborative links, nationally and internationally, with relevant services, community organisations, government agencies, universities, and research centres.

The established national partnership, spanning professional medical and nursing colleges, professional associations, leading health services, and government, is guided by cross-sector academic expertise. Together with co-design and co-production approaches, this structure reduces the risk of low organisational engagement and limited participant recruitment.

AWHL’s work is underpinned by robust methodology, including co-design and co-production approaches [28], evidence synthesis [26, 40, 41], and CFIR and RE-AIM frameworks [35, 39].

### Ethics and dissemination

Ethics approval was obtained from the Monash University Human Research Ethics Committee (Project ID: 25097) and research was conducted in accordance with the National Statement on Ethical Conduct in Human Research (2023) [46].

This protocol describes a prospectively planned implementation workstream within an ongoing, ethically approved national program of research. Findings will be disseminated through a variety of methods to maximise reach and engagement. This will include presentations of preliminary and final findings to individual stakeholder partners and their nominated committees and Boards, as well as presentations at national and international conferences. Manuscripts will be prepared for publication in open-access, peer-reviewed scientific journals to ensure the findings are rigorously presented and accessible to all.

## Discussion

### Rationale and approach to system-level change

Implementing and sustaining gender equity in healthcare leadership requires navigating complex, adaptive systems characterised by interacting organisational, policy, and workforce-level determinants. Implementing change within such systems presents significant challenges, particularly when aiming for sustainable and embedded transformation. The OCM workstream provides the strategic mechanism for aligning change efforts across national policy frameworks, organisational structures, and the everyday practices that shape leadership opportunities [47, 48].

### Implementation approach and methodological considerations

Progress depends on alignment across multiple levels – from national policy frameworks to organisational cultures and structures that shape leadership pathways. To accelerate gender equality in healthcare leadership, the AWHL initiative adopts a multi-level, co-produced approach, ensuring that stakeholders across all levels of the system are actively engaged in shaping and delivering change [28, 35]. Stakeholders will partner with the AWHL team to design, implement, evaluate, and scale interventions at the system, organisational, and individual levels. These interventions aim to measurably improve career progression for women in healthcare leadership.

The methodology integrates robust implementation science frameworks, co-design principles, and comprehensive data collection strategies [28, 35, 38]. At the systems level, both the outer setting – including national policies, legislation, funding mechanisms, regulatory bodies, unions, professional associations, and peak organisations – and the inner setting, such as health services and individual organisations, are considered. Interventions may include policy reform, awareness campaigns, support tools (e.g., measurement and evaluation frameworks), and educational opportunities, all designed to foster meaningful and lasting change for individuals within the system.

Without adequate support, strategic alignment, and leadership commitment, gender equality in healthcare leadership is unlikely to be achieved or sustained. Whole-of-system thinking is essential to drive meaningful progress. This requires consolidated efforts, prioritisation, resource allocation, and ongoing leadership commitment [26]. Optimising implementation demands evidence on the “who,” “what,” and “how,” as articulated through the LHS quadrants. This protocol outlines a national, systems-level initiative that responds to policy and stakeholder priorities by addressing: “who” (stakeholders across outer, inner, and individual levels), “what” (implementation toolkits, resources, and support mechanisms), and “how” (policies, processes, and strategies tailored to each level).

To our knowledge, this is the first national partnership of its kind and scale – bringing together strategic partners and key stakeholders, including women in the healthcare workforce, professional colleges and associations, leading health services, government agencies, and cross-sector academic experts. This partnership aims to enable coordinated action to understand, develop, and implement sustainable, evidence-informed system and organisational change to advance women in healthcare leadership.

### Implications for organisational practice and health system performance

The impact of AWHL’s OCM workstream will be assessed through both its reach – reflected in the number, diversity and engagement of partner organisations actively implementing change – and its effectiveness, assessed through changes in gender equity processes (e.g., maturity matrix scores [49]) and measurable gains in gender equality outcomes, such as those reported by the Commission for Gender Equality in the Public Sector in Victoria [50] and the Workplace Gender Equality Agency indicators in Australia [51]. Importantly, these indicators will be interpreted not merely as workforce or cultural metrics, but as leading indicators of health system performance – reflecting organisational environments that are more likely to support high-quality care, workforce retention, and equitable health outcomes. This also provides insight into how implementation science frameworks can be applied to workforce and organisational reform at scale, extending beyond traditional applications focused on clinical interventions.

### Innovation and transferability

While implementation of this study will occur within the Australian health system which has specific governance structures, regulatory settings and workforce arrangements that may differ from those in other countries, the implementation processes, determinant assessments, and strategy selection approaches described are transferable to other complex health systems, with adaptation to local context. The partnership model underpinning the AWHL initiative, together with the Monash Systems Change Approach used to drive evidence-informed reform, offers broad international applicability for addressing complex system change. While contextual factors will inevitably shape how specific interventions are implemented, the core principles, processes and frameworks used in this work are widely relevant and can inform comparable efforts across diverse settings.

Our approach applies rigorous organisational change management methodologies, underpinned by a shared vision, co-production, and a focus on measurable outcomes. Across all phases, we aim to accelerate the translation of evidence into sustainable organisational change. By applying and integrating established implementation science frameworks, this protocol strengthens understanding of how complex, system- and organisational-level change can be implemented, evaluated, and sustained – not only to gender equity initiatives, but to broader equity and leadership challenges across healthcare.

### Contribution to implementation science

This protocol advances implementation science by providing a replicable approach for operationalising systems-level organisational change through the integration of CFIR, Learning Health System, and RE-AIM. It demonstrates how determinant assessment, stakeholder co-production, adaptive implementation, and outcome evaluation can be aligned to support scale-up and sustainability in complex health systems.

Specifically, this protocol illustrates how determinant assessment (CFIR), iterative learning and adaptation (LHS), and structured evaluation (RE-AIM) can be integrated to guide the design, implementation, and evaluation of multi-level interventions. This alignment enables a dynamic and responsive implementation process, where strategies are informed by context, refined through continuous feedback, and evaluated across multiple dimensions of impact and sustainability.

Importantly, this work extends the application of implementation science beyond its more traditional focus on clinical interventions to encompass workforce and organisational transformation. The study addresses large-scale system- and organisational-level change, including policy implementation, culture change, and leadership development – areas that remain comparatively underdeveloped in the implementation science literature, despite their central role in shaping health system performance.

By embedding co-production, contextual tailoring, and cross-sector partnership within this integrated framework, this protocol also provides a transferable model for implementing and scaling complex interventions across diverse organisational settings. This approach offers practical and methodological insights for researchers and practitioners seeking to apply implementation science to large-scale, system-wide challenges in healthcare and beyond.

## Supplementary Information

Below is the link to the electronic supplementary material.

Supplementary Material 1

## Data Availability

No datasets were generated or analysed during the current study.

## Abbreviations

OCM: Organisational Change Management
AWHL: Advancing Women in Healthcare Leadership
CFIR: Consolidated Framework for Implementation Research
LHS: Learning Health System
RE-AIM: Reach, Effectiveness, Adoption, Implementation and Maintenance framework
MCHRI: Monash Centre for Health Research and Implementation
NHMRC: National Health and Medical Research Council
