# Supplementary material for: Implementing systems- and organisational-level change to advance gender equality in healthcare leadership: a mixed-methods implementation protocol
Source: Implement Sci Commun. 2026 Jun 26;7:187. doi: 10.1186/s43058-026-01030-w (PMC13579878; doi:10.1186/s43058-026-01030-w)
Supplement: Supplementary file 1 [file 43058_2026_1030_MOESM1_ESM.docx]

**SUPPLEMENTARY FILE 1**

**List of Partners of the Advancing Women in Healthcare Leadership (AWHL) national initiative**

1. Association of Australian Medical Research Institutes
2. Australian Academy of Health and Medical Sciences
3. Australian and New Zealand College of Anaesthetists
4. Australian College of Midwives
5. Australian College of Nursing
6. Australian Federation of Medical Women
7. Australian Medical Association (Lead Partner)
8. Australian National University
9. Australasian Nursing and Midwifery Clinical Trials Network
10. Cabrini Health
11. Eastern Health
12. Epworth HealthCare (Lead Partner)
13. The Fred Hollows Foundation
14. Metro South Health (Lead Partner)
15. Monash Health (Lead Partner)
16. Monash University
17. Royal Australasian College of Physicians
18. Royal Australasian College of Surgeons
19. Royal Australian College of General Practitioners (Lead Partner)
20. Science in Australia Gender Equity
21. The Australasian College of Dermatologists
22. The Royal Australasian College of Medical Administrators (Lead Partner)
23. The Royal Australian and New Zealand College of Gynaecologists
24. The Royal Australian and New Zealand College of Psychiatrists
25. University College Dublin
26. University of Glasgow
27. Victorian Department of Health
28. University of Warwick
